# Genetic evidence for a pathogenic role for the vitamin D3 metabolizing enzyme *CYP24A1* in multiple sclerosis^☆^

**DOI:** 10.1016/j.msard.2013.08.009

**Published:** 2014-03

**Authors:** Adaikalavan Ramasamy, Daniah Trabzuni, Paola Forabosco, Colin Smith, Robert Walker, Allissa Dillman, Sigurlaug Sveinbjornsdottir, North American Brain Expression Consortium (NABEC), UK Brain Expression Consortium (UKBEC), John Hardy, Michael E. Weale, Mina Ryten

**Affiliations:** a King's College London, Department of Medical & Molecular Genetics, Guy's Hospital, London SE1 9RT, UK; b Reta Lila Weston Research Laboratories, Department of Molecular Neuroscience, UCL Institute of Neurology, London WC1N 3BG, England, UK; c Department of Genetics, King Faisal Specialist Hospital and Research Centre, PO Box 3354, Riyadh 11211, Saudi Arabia; d Istituto di Genetica delle Popolazioni – CNR, Sassari, Italy; e Department of Neuropathology, MRC Sudden Death Brain Bank Project, University of Edinburgh, Wilkie Building, Teviot Place, Edinburgh EH8 9AG, Scotland, UK; f Laboratory of Neurogenetics, National Institute on Aging, National Institutes of Health, Bethesda, MD, USA; g Department of Neurology, MEHT, Broomfield Hospital, Court Road, CM1 7ET Essex, UK; h Department of Neurology, Queen Mary College, University of London, UK

**Keywords:** Multiple sclerosis, Genetics, Vitamin D, CYP24A1, Expression quantitative trait loci, Genome-wide association studies

## Abstract

**Background:**

Multiple sclerosis (MS) is a common disease of the central nervous system and a major cause of disability amongst young adults. Genome-wide association studies have identified many novel susceptibility loci including rs2248359. We hypothesized that genotypes of this locus could increase the risk of MS by regulating expression of neighboring gene, *CYP24A1* which encodes the enzyme responsible for initiating degradation of 1,25-dihydroxyvitamin D3.

**Methods:**

We investigated this hypothesis using paired gene expression and genotyping data from three independent datasets of neurologically healthy adults of European descent. The UK Brain Expression Consortium (UKBEC) consists of post-mortem samples across 10 brain regions originating from 134 individuals (1231 samples total). The North American Brain Expression Consortium (NABEC) consists of cerebellum and frontal cortex samples from 304 individuals (605 samples total). The brain dataset from Heinzen and colleagues consists of prefrontal cortex samples from 93 individuals. Additionally, we used gene network analysis to analyze UKBEC expression data to understand *CYP24A1* function in human brain.

**Findings:**

The risk allele, rs2248359-C, is strongly associated with increased expression of *CYP24A1* in frontal cortex (*p*-value=1.45×10^−13^), but not white matter. This association was replicated using data from NABEC (*p*-value=7.2×10^−6^) and Heinzen and colleagues (*p*-value=1.2×10^−4^). Network analysis shows a significant enrichment of terms related to immune response in eight out of the 10 brain regions.

**Interpretation:**

The known MS risk allele rs2248359-C increases *CYP24A1* expression in human brain providing a genetic link between MS and vitamin D metabolism, and predicting that the physiologically active form of vitamin D3 is protective. Vitamin D3's involvement in MS may relate to its immunomodulatory functions in human brain.

**Funding:**

Medical Research Council UK; King Faisal Specialist Hospital and Research Centre, Saudi Arabia; Intramural Research Program of the National Institute on Aging, National Institutes of Health, USA.

## Highlights

• SNP associated with MS is also associated with cortical CYP24A1 expression.
• CYP24A1 is the vitamin D metabolizing enzyme.
• This implies that those who have low brain levels of vitamin D for genetic reasons have increased risk of MS.
• This is consistent with epidemiological data on vitamin D and MS.

## 1 Introduction

Multiple sclerosis (MS) is one of the most common diseases of the central nervous system with a lifetime risk of 1:400 (Compston and Coles, 2002). It typically affects young adults causing intermittent neurological disturbances followed by progressive accumulation of disability. Studies of twins, siblings and adoptees demonstrate that MS has a significant heritable component, contributing to an overall familial recurrence rate of 20% (Hansen et al., 2005, Willer et al., 2003, Compston and Coles, 2008). Consistent with the understanding of MS primarily as an inflammatory disorder of the brain and spinal cord, the most established genetic associations with MS relate to the inheritance of the major histocompatibility complex and in particular the DR15 and DQ6 alleles.

More recently genome-wide association studies have provided additional insights. The largest collaborative genome-wide association study to date (Sawcer et al., 2011), involving nearly 10,000 cases and 17,000 controls derived from European-descent populations, highlighted a role for T cell-mediated immune mechanisms in MS, but also identified some novel susceptibility loci that appeared to be unrelated to the immune system. One example is the novel risk allele *rs2248359-C* (located on position 52,791,518 of chromosome 20, odds ratio of 1.12, reported *p*-value=2.50×10^−11^). This is a common variant (risk allele frequency estimate of 60% in Europeans) and is located less than 4 kilobases upstream of *CYP24A1* in a promoter associated region (as annotated within the Ensembl Regulatory Build), raising the possibility that it may operate by changing the expression of this gene, in effect making it an Expression Quantitative Trait Locus (eQTL). However, no functional evidence has been put forward to support this.

We test this hypothesis by investigating the effect of rs2248359 on gene expression in two large and independent brain consortiums – UK Brain Expression Consortium (UKBEC) (Trabzuni et al., 2011, Trabzuni et al., 2012, Ramasamy et al., 2013) and North American Brain Expression Consortium (NABEC) (International Parkinson's Disease Genomics Consortium (IPDGC), 2011, Gibbs et al., 2010, Hernandez et al., 2012) – as well a publicly available dataset (Heinzen et al., 2008).

## 2 Methods

### 2.1 UKBEC – tissue collection, RNA isolation and processing of brain samples analyzed using Affymetrix Exon 1.0 ST arrays

Brain samples were derived from 134 adult individuals of European ancestry with no significant neurological history or neuropathological abnormality (determined by a consultant neuropathologist). The samples were collected by the Medical Research Council (MRC) Sudden Death Brain and Tissue Bank (Millar et al., 2007), Edinburgh, UK, and the Sun Health Research Institute (SHRI) Brain Donation Program (Beach et al., 2008) an affiliate of Sun Health Corporation, USA. All samples had fully informed consent for retrieval and were authorized for ethically approved scientific investigation (Research Ethics Committee number 10/H0716/3).

Up to 10 brain regions were sampled from each individual resulting in a total of 1231 Affymetrix Human Exon 1.0 ST arrays (after quality control). The regions sampled were frontal cortex (FCTX), occipital cortex (specifically Brodmann area 17, OCTX), temporal cortex (TCTX), intralobular white matter (WHMT), hippocampus (HIPP), thalamus (THAL), putamen (PUTM), substantia nigra (SNIG), the inferior olivary nucleus (sub-dissected from the medulla, MEDU) and the cerebellar cortex (CRBL).

Total RNA was isolated from human post-mortem brain tissues using the miRNeasy 96 well kit (Qiagen, UK) and processed using the Ambion^®^ WT Expression Kit and Affymetrix GeneChip Whole Transcript Sense Target Labeling Assay, followed by hybridization to the Affymetrix Exon 1.0 ST Arrays according to the manufacturers' protocols. Hybridized arrays were scanned on an Affymetrix GeneChip^®^ Scanner 3000 7G and visually inspected for hybridization artefacts. Further details regarding tissue collection, sample demographics, RNA isolation, quality control and processing have been previously reported (Trabzuni et al., 2011).

### 2.2 NABEC – collection, RNA isolation and processing of brain samples analyzed using Illumina Human HT-12 v3 expression beadchip arrays

Cerebellar and frontal cortex samples originating from 304 neuropathologically-confirmed control adult individuals of European ancestry were collected as previously described (International Parkinson's Disease Genomics Consortium (IPDGC), 2011, Gibbs et al., 2010, Hernandez et al., 2012). Total RNA was extracted from dissected samples (100–200 mg) of human post-mortem brain tissue using a glass-Teflon homogenizer and 1 mL TRIzol (Invitrogen, Carlsbad, CA) according to the manufacturers' instructions. RNA was biotinylated and amplified using the Illumina^®^ TotalPrep-96 RNA Amplification Kit and directly hybridized onto Human HT12v3 Expression BeadChips (Illumina Inc., USA) in accordance with the manufacturer's instructions.

### 2.3 Brain dataset from Heinzen et al. (2008

This dataset consists of prefrontal cortex tissues from 93 adult individuals of European ancestry with no defined neuropshyciatric conditions. RNA was extracted using standard Qiagen protocols and hybridized onto Affymetrix Human Exon 1.0 ST arrays using standard Affymetrix protocols. The CEL files for the brain subset from this study were downloaded from Gene Expression Omnibus (GEO) website using the accession GSE30483

### 2.4 Pre-processing of expression profiles

Affymetrix Human Exon 1.0 ST arrays from UKBEC were pre-processed using Robust Multi-array Average (quantile normalization, probeset summary by median polish) algorithm in Affymetrix Power Tools 1.14.3 software (http://www.affymetrix.com/partners_programs/ programs/developer/tools/powertools.affx). After re-mapping the Affymetrix probe sets onto human genome build 19 (GRCh37) as documented in the Netaffx annotation file (HuEx-1_0-st-v2 Probeset Annotations, Release 31), we restricted analysis to probe sets that had gene annotation and contained at least three probes that were uniquely hybridized and did not contain any polymorphism (Ramasamy et al., 2013). This resulted in 14 exon-level probesets for *CYP24A1* (Affymetrix transcript ID 3910429) from which we calculated the transcript-level expression as the 10% Winsorized mean. We pre-processed the expression data for the brain subset of Heinzen et al. (2008) in the same way. Prior to use in eQTL analyses, the expression data was corrected for gender and batch effects (date of hybridization and brain bank or collection sites), as described previously (Trabzuni et al., 2011).

The expression data from Illumina Human HT12v3 Expression BeadChip Arrays from NABEC were analyzed using the Gene Expression Module 3.2.7 within Illumina^®^ BeadStudio. Raw intensity values for each probe were transformed using the cubic spline normalization method and then log2 transformed for mRNA analysis. We re-mapped the annotation for probes according to ReMOAT (Barbosa-Morais et al., 2010) on the human genome build 19 and then restricted the analysis to genes that were reliable, uniquely hybridized and were associated with gene descriptions. The probe for *CYP24A1* on this array is ILMN_1685663. Prior to use in eQTL analyses, the expression data was corrected for gender, age, PMI and batch effects (hybridization batch effects and brain bank), as described previously (Gibbs et al., 2010).

### 2.5 DNA extraction, genotyping of rs2248359 and imputation

Genomic DNA for individuals from UKBEC was extracted from sub-dissected samples (100–200 mg) of human post-mortem brain tissue using Gentra Puregene Kit (Qiagen, UK). Samples from every individual were run on two different genotype chips: the Illumina Infinium Omni1-Quad BeadChip and the ImmunoChip, a custom genotyping array designed for the fine-mapping of auto-immune disorders (International Parkinson's Disease Genomics Consortium (IPDGC), 2011, Nalls et al., 2011). The BeadChips were scanned using an iScan (Illumina, USA) with an AutoLoader (Illumina, USA). GenomeStudio v.1.8.X (Illumina, USA) was used for analyzing the data and generating SNP calls. Three individuals suspected of being of non-European ancestry were identified in UKBEC (134 remaining) using principal components projection and excluded from analysis. The DNA for individuals from NABEC were extracted and analyzed in similarly using the Illumina Infinium HumanHap550 v3 (Illumina, USA). The DNA for Heinzen et al. (2008) was extracted and analyzed using standard Qiagen protocols using the Illumina Human Hap550K chips.

The risk SNP rs2248359 was genotyped in all three datasets. To explore the presence of other eQTL signals, we can use imputation to infer additional untyped SNPs by using the identified linkage disequilibrium information between typed and untyped SNPs from reference panels that have a vastly more comprehensive set of SNPs. We used the software MaCH (Li et al., 2010, Li et al., 2009) and minimac (http://genome.sph.umich.edu/wiki/Minimac) with the 1000 Genomes (March 2012 release) as reference panel to impute the UKBEC and NABEC datasets. We used the resulting SNPs with good post-imputation quality (*r*^2^>0.50) and minor allele frequency of at least 5% in subsequent analyses.

### 2.6 Statistical analysis for identifying eQTL

The association between gene expression residuals and SNP was tested using a linear regression model in R software (http://www.r-project.org/) assuming an additive genetic model for genotypes. Unless stated otherwise, the reported *p*-values are nominal *p*-values.

### 2.7 Weighted Gene Co-expressed Network Analysis (WGCNA) of the UKBEC gene expression dataset

Analysis of co-expression of genes is particularly sensitive to the presence of outlier samples. Therefore, we first applied rigorous quality control procedures on the UKBEC samples from the MRC Sudden Death Brain and Tissue Bank to ensure the highest possible level of quality. Outliers were identified by repeated visual inspection of the hierarchical clustering of samples with Euclidean distance and the inter-array correlation metric. We also restricted to 15,409 transcripts (measured as 10% windsorized mean) that passed the detection above background criteria (*p*-value<0.001 in at least 50% of samples in at least one brain region), had a coefficient of variation >5% and at least one expression value exceeding five in at least one brain region (Forabosco et al., 2013).

The WGCNA network (Langfelder and Horvath, 2008) was constructed for each tissue separately using a signed network with power (Beta) of 12 to achieve a scale-free topology. A dissimilarity matrix based on Topological Overlap Measure (TOM), a pairwise measure of node similarity (i.e. how the neighbors of a gene are to the neighbors of another gene) was used to identify gene modules (i.e. densely interconnected and co-expressed genes) which has been shown to placing functionally related genes into groups.

Module preservation statistics calculate how well a module from one tissue (reference data) is reproducible (or preserved) in another tissue (test data). To construct a statistical measure of preservation, one may use a simple cross-tabulation analysis of module memberships and related Fisher exact test *p*-value, or use a more refined preservation statistics that do not use the module assignment in the test data (so called network-based statistics). Network-based statistics assess whether the modules features defined in a reference data are preserved in a test dataset. Here we report the Z summary statistic that aggregates different preservation statistics (Langfelder et al., 2011), and assess both whether module nodes remain highly connected in the test network (quantify density), and compares the connectivity pattern between nodes in the reference network to test network (quantify connectivity pattern). In order to determine whether the Z summary is higher than expected by chance, we used a permutation test procedure implemented in a WGCNA function, which randomly permutes the module assignment in the test data, and calculates the mean and variance of the preservation statistic under the null hypothesis of no relationship between the module assignments in reference and test data. Then, by standardizing each observed preservation statistic with regard to the mean and variance, we use the thresholds proposed by (Langfelder et al., 2011): Zsummary <2 implies no evidence for module preservation, 2<Zsummary<10 implies weak to moderate evidence, and Zsummary >10 implies strong evidence for module preservation.

### 2.8 Gene set enrichment analysis with DAVID

To evaluate the biological and functional relevance of co-expressed genes within the *CYP24A1*-containing modules, we used DAVID v6.7 (http://david.abcc.ncifcrf.gov/), the database for annotation, visualization and integrated discovery (Huang da et al., 2009). We examined the over-representation (i.e. enrichment) of Gene Ontology (GO) terms amongst the genes within each module by comparing numbers of significant genes annotated within a given biological category as compared with chance.

## 3 Results

### 3.1 Distribution of *CYP24A1* expression across the human brain and evidence for regulation by rs2248359

To investigate the impact of the MS risk SNP rs2248359 on gene expression in human brain we used paired gene expression and genotyping data generated by UKBEC (Trabzuni et al., 2011, Trabzuni et al., 2012, Ramasamy et al., 2013). This dataset is based on samples originating from 134 individuals of European descent. In all cases there was no history of a neurological disorder and control status was confirmed by histology with assessment by a neuropathologist. The individuals sampled had a mean age at death of 59 years old (range 16–102), were mostly men (74.5%) and the main cause of death was ischemic heart disease (44.7%). For each individual, up to 10 anatomical brain regions were sampled (for a total of 1231 arrays) to provide genome-wide expression data, including information on *CYP24A1* expression. The brain regions analyzed includes those commonly affected in MS, namely subcortical white matter.

**Fig. 1:**
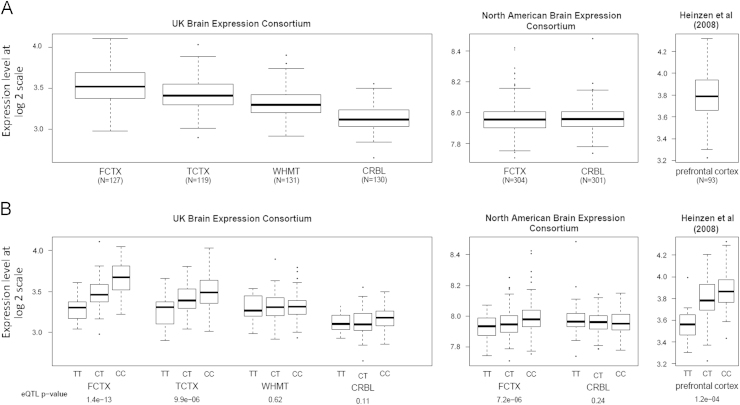
Regional and stratified plot. (A) Boxplot showing the distribution of mRNA levels across the three datasets (selected tissues from UKBEC). Whiskers extend from the box to 1.5 times the inter-quartile range. (B) mRNA levels stratified by the genotypes of the MS risk SNP rs2248359 (selected tissues from UKBEC). The eQTL *p*-values are given on the last *x*-axis.

We demonstrate widespread expression of *CYP24A1* mRNA in the human brain (Fig. 1A, Supplementary Fig. 1A). However, when we stratify mRNA levels by rs2248359 genotypes, we find very strong evidence for the association between the risk allele C and increased expression of *CYP24A1*. This association is evident in frontal cortex (*p*-value=1.45×10^−13^) and temporal cortex (*p*-value=9.93×10^−6^), but not in subcortical white matter (Fig. 1B) or any other regions in the UKBEC dataset (Supplementary Fig. 1B). A mixed model approach with brain regions and individuals as random effects covariates showed significant heterogeneity (*p*=0.001) between the brain regions.

Similarly, we find a significant association between rs2248359 and *CYP241* expression in frontal cortex (*p*-value=7.2×10^−6^; Fig. 1B) but not cerebellum in the NABEC dataset (Fig. 1B). Finally, Heinzen et al. (2008 dataset also shows an association in the prefrontal cortex samples (*p*-value=1.2×10^−4^, Fig. 1B). The direction of association was consistent across all three datasets and there was no evidence for associations with any other neighboring genes.

### 3.2 Refining eQTL signals in the genomic region

**Fig. 2:**
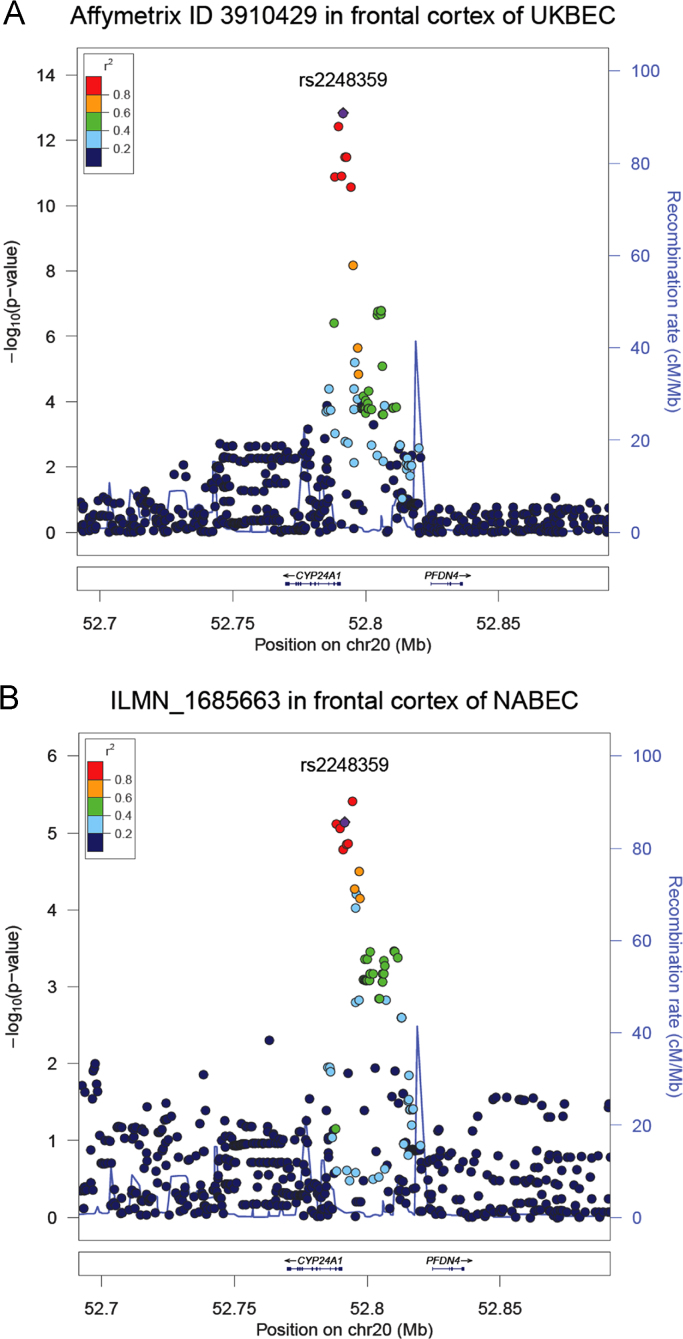
Refining eQTL signals in the genomic region. Regional association plot illustrating the expression quantitative trait loci (eQTL) around the gene *CYP24A1* in frontal cortex for (A) UKBEC dataset and (B) NABEC dataset. The MS risk SNP rs2248359 is shown in purple and the LD measures are with respect to this SNP.

We investigated the genomic region around rs2248359 for other SNPs capable of regulating *CYP24A1* expression in frontal cortex by imputing the genotype data for UKBEC and NABEC. We considered all SNPs within 1 Mb of the transcription start and stop site for this gene (Fig. 2). This analysis confirms that there is only one eQTL signal in this region for each dataset. In the UKBEC dataset, the best signal was indeed the risk variant under investigation (rs2248359 which is shown as a purple circle in Fig. 2A). In the NABEC dataset, the best signal was conferred by rs2104134 (Fig. 2B, red circle) which is located 2,932 bases upstream of the risk variant rs2248359 and in high linkage disequilibrium with it (haplotype *r*^2^=0.91). However, we note that rs2104134 is imputed while rs2248359 is genotyped and the difference in statistical significance is marginal (*p*-value of 3.9×10^−6^ vs. 7.2×10^−6^). Even though we did not impute Heinzen et al. (2008) dataset, we can confirm that the eQTL with rs2248359 was again the most significant signal amongst genotyped SNPs.

### 3.3 Insights into the function of *CYP24A1* in human brain using network analysis

We investigated the role of *CYP24A1* using Weighted Gene Co-Expression Network Analysis (WGCNA) to analyze human brain expression data from UKBEC (Forabosco et al., 2013). This approach groups genes into modules in an unsupervised manner (Langfelder and Horvath 2008) and has been shown to be extremely useful in the identification of modules of biologically related genes that are co-expressed (Rosen et al., 2011, Oldham et al., 2008). Using this approach we identified a *CYP24A1*-containing module of genes in all 10 of the brain regions analyzed. This module was large in all cases (average number of genes=4300) and was well preserved across the brain (Z summary preservation values >20 across all pairwise tissues, Supplementary Fig. 2). Using Module Membership (MM), as defined by how well the expression pattern of each gene within a module correlates with the first principal component of gene expression for that module, we assessed the extent to which *CYP24A1* “belongs” to each module. The MM for *CYP24A1* was above the median in nine out of 10 brain regions (minimum=47th quantile, maximum=68th), indicating strong membership.

To evaluate the biological and functional relevance of genes within the *CYP24A1*-containing modules we used Gene Ontology (GO) enrichment analysis. Focusing on GO terms relating to biological processes, this analysis demonstrated a significant over-representation of genes annotated to the “immune response” term (minimum Bonferroni-corrected *p*-value=1.80×10^−11^, Table 1) within the *CYP24A1*-containing modules in eight of the 10 brain regions analyzed, the exceptions being temporal cortex and white matter. In fact, “immune response” was the most significant term in this category on the basis of corrected *p*-values. In keeping with this finding similar analyses focused on the enrichment of canonical gene sets defined by the Kyoto Encyclopedia of Genes and Genomes (KEGG) and relating to known pathways identified the cytokine-cytokine receptor interaction pathway in eight out of 10 brain regions (minimum Bonferroni-corrected *p*-value=5.45×10^−5^).

**Table 1:** Summary of the results of GO enrichment analysis performed on the *CYP24A1*-containing modules in each brain region with all biological process terms with a Bonferroni-corrected *p*-value of<0.05 in at least five brain regions reported. Color coding reflects (*p*) value.

## 4 Discussion

In this paper, we used three independent post-mortem human brain expression datasets to show that the risk allele C of a known MS disease SNP rs2248359 is strongly associated with increased expression of *CYP24A1*. The increase in expression could be a direct effect of the risk SNP or could be an effect of a SNP in linkage disequilibrium with this SNP.

However, the SNP identified is in fact located within a genomic region predicted by ENCODE to be a promoter based on a range of experiments (DNAse I hypersensitivity site identification amongst others) performed in H1ESC and HepG2 cells. We would therefore predict that the SNP changes the efficiency of the CYP24A1 promoter resulting in changes in transcriptional rate and mRNA levels.

We note that in UKBEC, rs2248359 had the greatest impact on *CYP24A1* expression in frontal cortex and temporal cortex despite the gene being widely expressed in human brain and MS being classically characterized as a disease of white matter. Although this is surprising, this finding is in keeping with the increasing recognition of the extensive involvement of gray matter in MS pathology (Geurts et al., 2012). Demyelination in the gray matter commonly occurs and in fact gray matter pathology is more closely associated with neurological and neuropsychological disability than white matter lesions or whole brain atrophy (Geurts et al., 2012).

Given that *CYP24A1* encodes the enzyme responsible for initiating the degradation of 1,25-dihydroxyvitamin D3 (the physiologically active form of vitamin D3) we suggest that the observation of an eQTL provides genetic evidence for a pathogenic role for low levels of 1,25-dihydroxyvitamin D3 in MS. Previous genetic evidence for such a link has been inconsistent. One study (Ramagopalan et al., 2011) reported that rare, loss-of-function variation in *CYP27B1* (the gene encoding 25-hydroxyvitamin D 1α hydroxylase, which converts 25 hydroxyvitamin D(25[OH]D) into its active form) increased MS risk, but two subsequent studies (Barizzone et al., 2013, Ban et al., 2013) failed to replicate this finding. However, the link between MS and vitamin D is supported by epidemiological evidence (Simon et al., 2012). In fact, the possibility that vitamin D deficiency may be a risk factor for multiple sclerosis was proposed more than 30 years ago, primarily to explain the observation of a latitude gradient in MS prevalence (Simon et al., 2012). This has led to further studies demonstrating that low vitamin D levels are associated with a high risk of developing MS and vitamin D supplementation reduces the risk (Simon et al., 2012).

With regard to how vitamin D might exert a protective effect on MS risk, there is a growing body of work to suggest that vitamin D has immunomodulatory functions which could be relevant to pathogenesis (Simon et al., 2012, Ascherio et al., 2012). This hypothesis is supported by the results of gene expression network analysis on human brain tissue and in particular the enrichment of immune-related genes within the *CYP24A1*-containing gene modules. It is noteworthy that this enrichment was not observed in the *CYP24A1*-containing gene module in white matter, raising the possibility that there may be some differences in the underlying pathogenic processes in MS in white versus gray matter.

Thus, in summary we demonstrate that the known MS risk SNP rs2248359 regulates *CYP24A1* expression in human brain providing a genetic link between MS and vitamin D metabolism. We also provide evidence in support of the hypothesis that the protective effects of vitamin D are related to its immunomodulatory functions in human brain. Further research into this proposed mechanism is warranted and the use of dietary supplementation should, perhaps, be considered.

## Sources of funding

This work was supported by the MRC through the MRC Sudden Death Brain Bank (C.S.) and by a Project Grant (G0901254 to J.H. and M.W.) and Training Fellowship (G0802462 to M.R.). D.T. was supported by the King Faisal Specialist Hospital and Research Centre, Saudi Arabia.

The work performed by the North American Brain Expression Consortium was supported in part by the Intramural Research Program of the National Institute on Aging, National Institutes of Health, part of the US Department of Health and Human Services; Project number ZIA AG000932-04.

We are grateful to the Banner Sun Health Research Institute Brain and Body Donation Program of Sun City, Arizona for the provision of human biospecimens. The Brain and Body Donation Program is supported by the National Institute of Neurological Disorders and Stroke (U24 NS072026 National Brain and Tissue Resource for Parkinson's Disease and Related Disorders), the National Institute on Aging (P30 AG19610 Arizona Alzheimer's Disease Core Center), the Arizona Department of Health Services (contract 211002, Arizona Alzheimer's Research Center), the Arizona Biomedical Research Commission (contracts 4001, 0011, 05-901 and 1001 to the Arizona Parkinson's Disease Consortium) and the Michael J. Fox Foundation for Parkinson's Research.

The funders had no role in study design, data collection and analysis, decision to publish or preparation of the manuscript.

## Author contributions

AR data analysis, DT labwork and data analysis, PF network analysis, CS pathological analysis, RW brain dissection and documentation, AD labwork, JH study design and funding, MEW study design and funding, MR study design, funding and drafting primary manuscript. All authors, manuscript critique and redrafting.

## Conflicts of interest

The authors have no potential conflicting financial, personal or professional interests.

## Footnotes

Appendix A Supplementary data associated with this article can be found in the online version at doi:10.1016/j.msard.2013.08.009.
