# Supplementary figures and images for: Genetic evidence for a pathogenic role for the vitamin D3 metabolizing enzyme CYP24A1 in multiple sclerosis
Source: Mult Scler Relat Disord. 2014 Mar;3(2):211–9. doi: 10.1016/j.msard.2013.08.009 (PMC4278441; doi:10.1016/j.msard.2013.08.009)

# Supplementary Figure 1

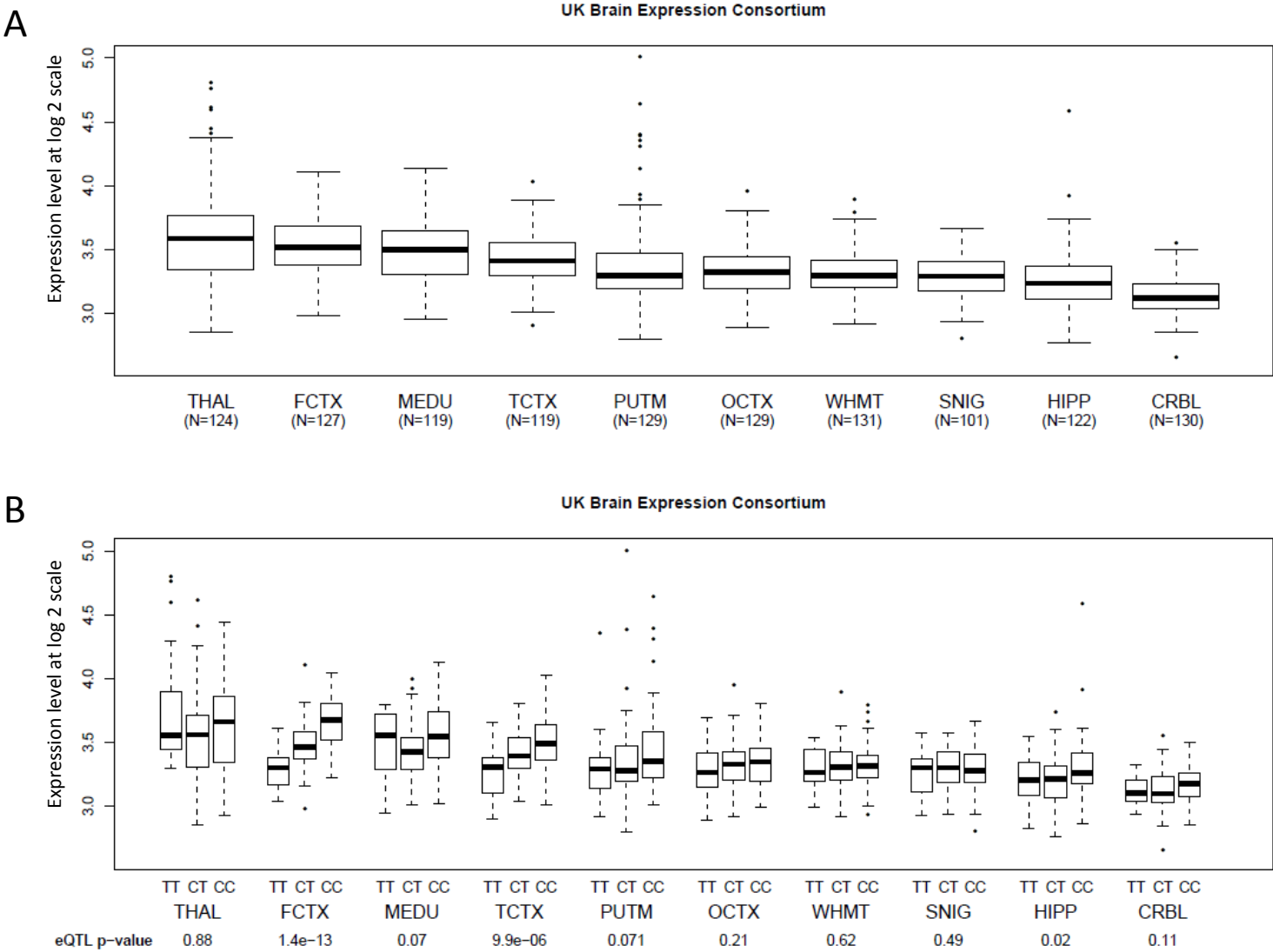

Supplement: Supplementary file 1 — Supplementary material [file mmc1.pdf]

# Supplementary Figure 2

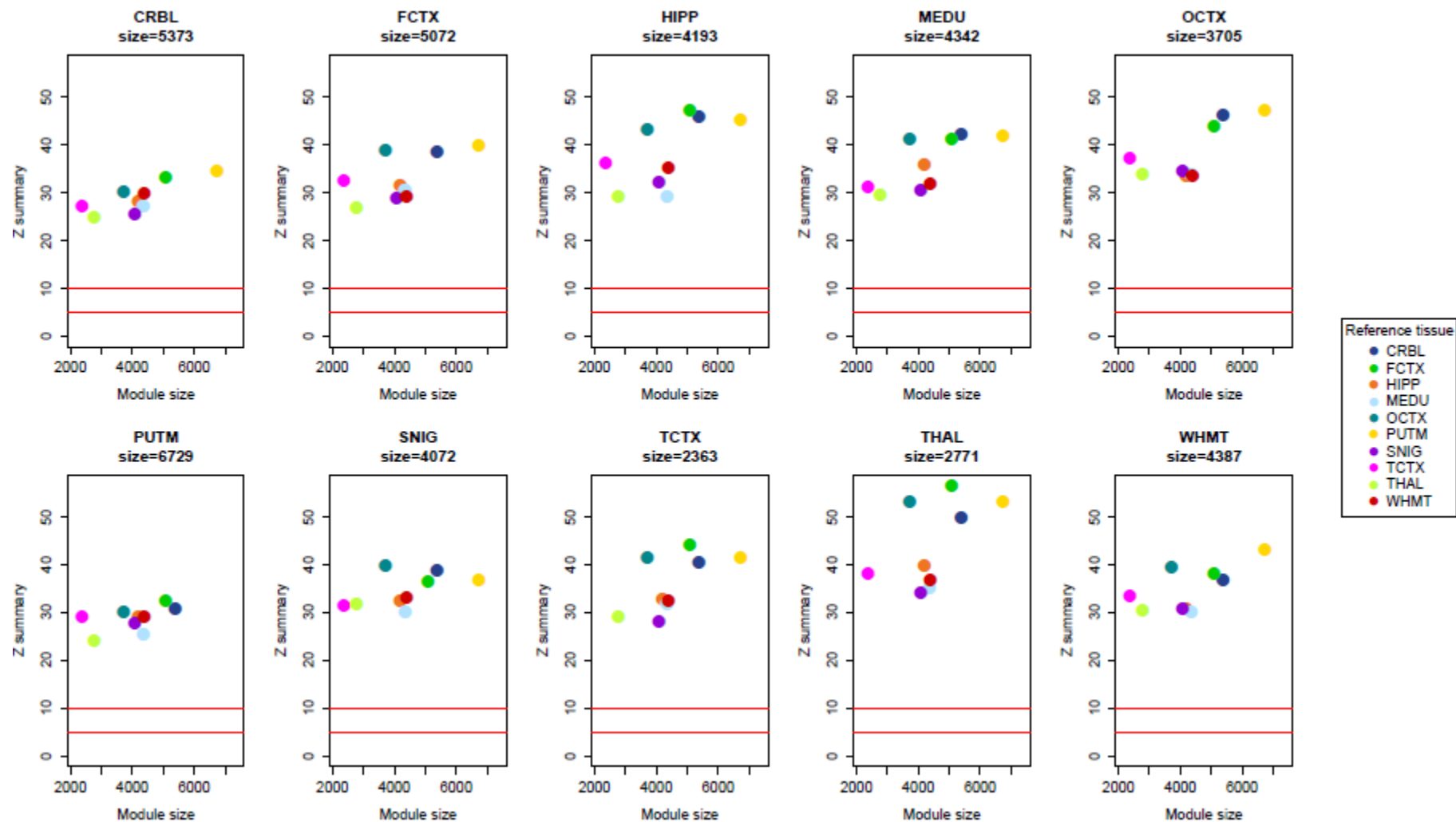

Supplement: Supplementary file 2 — Supplementary material [file mmc2.pdf]
